# Genetic and morphological variants of *Acidovorax avenae* subsp. *avenae* cause red stripe of sugarcane in China

**DOI:** 10.3389/fpls.2023.1127928

**Published:** 2023-02-06

**Authors:** Jian-Ying Zhao, Juan Chen, Zhong-Ting Hu, Juan Li, Hua-Ying Fu, Philippe C. Rott, San-Ji Gao

**Affiliations:** ^1^ National Engineering Research Center for Sugarcane, Fujian Agriculture and Forestry University, Fuzhou, Fujian, China; ^2^ CIRAD, UMR PHIM, Montpellier, France; ^3^ PHIM Plant Health Institute, Univ Montpellier, CIRAD, INRAE, Institut Agro, IRD, Montpellier, France

**Keywords:** *Acidovorax avenae* subsp. *avenae*, genetic diversity, multilocus sequence typing, pathogenicity, red stripe, *Saccharum* spp., salicylic acid

## Abstract

Sugarcane (*Saccharum* spp.) is an important cash crop for production of sugar and bioethanol. Red stripe caused by *Acidovorax avenae* subsp. *avenae* (*Aaa*) is a disease that occurs in numerous sugarcane-growing regions worldwide. In this study, 17 strains of *Aaa* were isolated from 13 symptomatic leaf samples in China. Nine of these strains produced white-cream colonies on nutrient agar medium while the other eight produced yellow colonies. In pairwise sequence comparisons of the 16S-23S rRNA internally transcribed spacer (ITS), the 17 strains had 98.4-100% nucleotide identity among each other and 98.2-99.5% identity with the reference strain of *Aaa* (ATCC 19860). Three RFLP patterns based on this ITS sequence were also found among the strains of *Aaa* obtained in this study. Multilocus sequence typing (MLST) based on five housekeeping genes (*ugp*B, *pil*T, *lep*A, *trp*B, and *glt*A) revealed that the strains of *Aaa* from sugarcane in China and a strain of *Aaa* (30179) isolated from sorghum in Brazil formed a unique evolutionary subclade. Twenty-four additional strains of *Aaa* from sugarcane in Argentina and from other crops worldwide were distributed in two other and separate subclades, suggesting that strains of *A. avenae* from sugarcane are clonal populations with local specificities. Two strains of *Aaa* from China (CNGX08 forming white-cream colored colonies and CNGD05 forming yellow colonies) induced severe symptoms of red stripe in sugarcane varieties LC07-150 and ZZ8 but differed based on disease incidence in two separate inoculation experiments. Infected plants also exhibited increased salicylic acid (SA) content and transcript expression of gene *PR-1*, indicating that the SA-mediated signal pathway is involved in the response to infection by *Aaa*. Consequently, red stripe of sugarcane in China is caused by genetically different strains of *Aaa* and at least two morphological variants. The impact of these independent variations on epidemics of red stripe remains to be investigated.

## 1 Introduction

Bacterial species of *Acidovorax* (family *Comamonadaceae*) are gram-negative beta-proteobacteria that can be separated in two major groups. One group is formed by four non-plant species (*A. delafieldii*, *A. defluvii*, *A. facilis*, and *A. temperans*) isolated from environmental and clinical sources, while the other group includes three plant pathogenic species (*A. avenae*, *A. cattleyae*, and *A. citrulli*) (Willems and Gillis, 2015). Additional species of *Acidovorax* are commensal bacteria or plant growth-promoting bacteria (Siani et al., 2021); for example, *A. radicis* (strain N35) isolated from wheat roots increases the growth of barley cultivars and reduces aphid density (Zytynska et al., 2020). A list of 15 *Acidovorax* species, including three subspecies, was recently established (De Vos et al., 2018). In the 2015 Bergey’s Manual of Systematic Bacteriology, *A. avenae* includes three subspecies that cause economically important diseases in a wide range of plants (Willems and Gillis, 2015). Each of these subspecies has a different host range. *A. avenae* subsp. *citrulli* (*Aac*) infects plants of the *Cucurbitaceae*, while *A. avenae* subsp. *cattleyae* (*Aaca*) infects only orchid species of the genera *Cattleya* and *Phalaenopsis*. The third subspecies, *A. avenae* subsp. *avenae* (*Aaa*), causes bacterial blight or red/brown stripe diseases in a variety of plants of the *Gramineae* (including barley, creeping bentgrass, maize, millet, oat, rice, rye, sorghum, sugarcane, and vasey grass), the *Theaceae* (tea), and the *Strelitziaceae* (white bird of paradise) (Willems and Gillis, 2015; Li et al., 2018; Fontana et al., 2019).

Sugarcane (*Saccharum* spp.) is the most important sugar crop, accounting for 80% and 90% of the sugar production in the world and in China, respectively (Liu et al., 2022). Red stripe caused by *Aaa* has been reported in more than 60 countries and is one of three main bacterial diseases of sugarcane distributed worldwide (Rott and Davis, 2000). Leaf stripes and stalk top rot are two types of symptoms of sugarcane red stripe. These symptoms can appear separately or simultaneously in the field (Martin et al., 1989; Rott and Davis, 2000; Fu et al., 2017). The disease, and especially the top rot form, can result in significant yield losses. In Argentina, red stripe recently affected 30% of the millable stems, thus causing economic losses (Fontana et al., 2019). Disease incidences greater than 50% were observed in variety CoJ 85 in India and in variety COLMEX 9408 in Mexico (Bipen et al., 2014; Hernández-Juárez et al., 2021). During the last decade, red stripe outbreaks occurred in two main sugarcane-growing provinces in China. In the Guangxi province, disease incidences in the field reached 23% in variety FN38 (Fu et al., 2017), 39% in BT15-173 (Li et al., 2021), 50% in GT58, and 22% in LC05-136 (Li et al., 2022). In the Yunnan province, 80% of plants of variety YZ03-194 were symptomatic (Shan et al., 2017).

Efficient pathogen diagnostic and genotyping methods are needed for epidemiological surveillance and disease management (Bertani et al., 2021; Strachan et al., 2022). Besides microbiological methods, molecular typing methods have contributed to accurate identification and characterization of *Aaa* worldwide. In Mexico, molecular identification of *Aaa* was performed using 16S rDNA sequences of strains of the pathogen from three sugarcane-producing agroecological regions (Hernández-Juárez et al., 2021). The pathogen was identified in Argentina using PCR and primers Oaf1/Oar1 targeting the 16S–23S rDNA ITS (internally transcribed spacer) region (Fontana et al., 2013). Presence of at least four different genotypes and ten phylogenetic groups of *Aaa* was reported in this country using random amplified polymorphic DNA (RAPD) markers and repetitive element polymorphism-based polymerase chain reaction (rep-PCR), respectively (Bertani et al., 2021). Furthermore, multilocus sequence typing (MLST) of *Aaa* strains from different hosts revealed that a novel clonal group of strains was causing red stripe of sugarcane in Argentina (Fontana et al., 2019). Five major groups of *Aaa* strains were found in China by restriction fragment length polymorphism (RFLP) analysis (Li et al., 2018).

Multiple genes and proteins and their related metabolites and signal pathways are involved in sugarcane response to *Aaa* infection, as revealed by transcriptomic (Santa et al., 2016; Thiebaut et al., 2017; Chu et al., 2020), proteomic (Zhou et al., 2021), and genome-wide analyses (Zhou et al., 2021; Chu et al., 2022). Several genes related to signal transduction by salicylic acid (SA) are involved in sugarcane resistance to *Aaa*, but their specific role remains to be investigated (Chu et al., 2020; Chu et al., 2022). The first objective of this study was to characterize isolates of *Aaa* from China using conventional microbiological and pathogenicity assays, as well as MLST. The second objective was to investigate the importance of SA-medicated resistance gene *PR-1* (coding for pathogenesis-related protein 1) in response of sugarcane to infection by two morphologically different strains of *Aaa*.

## 2 Materials and methods

### 2.1 Leaf sample collection

From May to July 2021, 13 sugarcane leaves with red stripe symptoms were collected in sugarcane fields of the provinces of Guangdong and Guangxi in China (**Table S1**). The symptoms of the diseased samples were photographed on site and taken to the laboratory for isolation and identification of the causal agent(s) (**Figure S1**).

### 2.2 Isolation and molecular diagnosis of pathogenic bacteria

The isolation and purification of bacteria was performed using the agar-streak method. Briefly, symptomatic leaves were first disinfected for 1 min with 75% alcohol and then rinsed three times with sterile water. At the junction of the diseased and healthy leaf tissue, two or three fragments (2-3 mm × 4-5 cm) were cut into small pieces that were introduced in a 2 mL-microtube. This microtube contained one 4 mm-steel ball, two 2 mm-steel balls, and 100 µL of sterile water. Leaf tissue was homogenized for 60 s with a MM400 beater (Verder Shanghai Instruments and Equipment Co., Ltd, Shanghai, China). Subsequently, 400 µL of sterile water were added to each tube. After a 30 min incubation, the supernatant of each tube was spread on nutrient agar (NA) medium using the quadrant streak method. Agar plates were then incubated at 30°C for 12-16 h. Bacterial colonies with a yellow or a white color were isolated, purified, and tested by PCR using *Aaa*-specific primers RS-ITS-F1 and RS-ITS-R1, as described by Li et al. (2018). The PCR program was 3 min at 94°C for initial denaturation; 35 cycles of 45 s at 94°C for denaturation, 30 s at 58°C for annealing, and 45 s at 72°C for extension; and 10 min at 72°C for final extension. All PCR-positive colonies were stored on NA plates at 4°C and in 20% glycerol at -80°C until further investigations. Five colonies of 24 hour-old bacteria growing on NA medium were randomly selected for each strain to measure their colony size.

### 2.3 Morphological observation of bacterial colonies

Freshly isolated and 24-hour-old single colonies of *Aaa* CNGX08 (white) and CNGD05 (yellow) were suspended in 50 μL sterile H_2_O. A loop of bacterial suspension was placed on a copper mesh for 1 min and excess of liquid was removed with a filter paper. Bacteria were stained with a solution of 2.5% phosphor-tungstic acid (pH 7.0) for 3 s. Morphology of bacterial cells was observed with a HT-7700 transmission electron microscope (Hitachi High-Tech, Shanghai, China) and digital images were acquired with an AMT CCD camera (Hitachi High-Tech).

### 2.4 Bacterial genomic DNA extraction

Bacterial genomic DNA was extracted using the Bacterial Genome DNA Extraction Kit (Tiangen, Beijing, China). DNA quality and quantity tests were performed using 1% agar gel electrophoresis and the SynergyTM H1 multifunctional microplate reader (BioTek, Vermont, USA). The DNA samples were stored at -80°C until further use.

### 2.5 PCR amplification of housekeeping genes

Five housekeeping genes (*ugp*B, *pil*T, *lep*A, *trp*B, and *glt*A) were amplified by PCR using primer pairs reported by Feng et al. (2009). The 50 μL reaction mix included 1.0 μL bacterial genomic DNA (100 ng/μL), 20 μL Green Taq Mix (Vazyme Biotech, Nanjing, China), 1.0 μL each of forward and reverse primers (10 μmol/L), and 27 μL sterile water. The PCR program was 5 min at 95°C for initial denaturation; 35 cycles of 30 s at 95°C for denaturation, 30 s at 60°C for annealing, and 30 s at 72°C for extension; and 5 min at 72°C for final extension. The expected amplification product had a size of 444, 398, 489, 434, and 481 bp for *ugp*B, *pil*T, *lep*A, *trp*B, and *glt*A, respectively. Bacterial genomic DNA of *Aaa* strain ATCC 19860 (Institute of Microbiology, Chinese Academy of Sciences) was used as the positive control. Sterile distilled water was used as the blank control. All primer information is given in **Table S2**.

### 2.6 Cloning of PCR products

All the PCR products (sections 2.2 and 2.5) were purified and then cloned into the pMD19-T vector. Three positive clones per PCR product were randomly selected for sequencing by Sangon Biotech (Shanghai, China). Sequences were deposited at the NCBI GenBank database under accession numbers OP738811-OP738827 for the 16S-23S rDNA ITS region, and under accession numbers ON707276-ON707358 and OP747511-OP747512 for the five house-keeping genes.

### 2.7 RFLP, MLST and nucleotide identity analysis

A restriction fragment length polymorphism (RFLP) analysis of the *Aaa* sequences obtained with primers RS-ITS-F1/RS-ITS-R1 was performed *in silico* using restriction enzymes *Hin*dIII and *Eco*RI with DNAMAN software (Lynnon Biosoft, San Ramon, USA). The restriction patterns were identified and labelled as described by Li et al. (2018).

A concatenated sequence of the five housekeeping genes mentioned in section 2.5 was produced for 54 bacterial strains: 42 *Aaa* strains (17 obtained in this study and 25 retrieved from the NCBI library), 10 *Aac* strains, one *Aaca* strain, and one *A. facilis* strain (used as outgroup strain). The 54 concatenated sequences were aligned using the ClustalW algorithm in the software MEGA 11 (Tamura et al., 2021). A phylogenetic tree was constructed using the neighbor-joining (NJ) method with 1000 bootstrap replicates. Sequence identity analysis was performed with BioEdit V7.0.9.0 (Borland, Scotts Valley, USA). A heat map was produced with SDTv1.2 (The University of Cape Town, South Africa). The characteristics of all tested strains of *Acidovorax* are given in **Table S1**.

### 2.8 Pathogenicity assay of two bacterial strains

Stalk cuttings with a single bud each were prepared from healthy sugarcane varieties LC07-150 (source of *Aaa* strain CNGX08) and ZZ8 (source of *Aaa* strain CNGD05). Cuttings were planted in pots (5.5 x 4 x 6 cm) filled with nutrient soil (Pindstrup Mosebrug A/S, PINDSTRUP, Denmark) and grown in a climate chamber at 30°C with 16 h light/8 h night and 65% humidity. When plants had 1-2 fully expanded leaves, they were inoculated using the leaf puncture method. Briefly, the upper side of the leaf was punctured but not pierced with a disposable syringe and the wounded area was wrapped with sterile cotton. One mL of bacterial suspension (10^8^ CFU/mL) was placed on the cotton for inoculation with a single strain of the pathogen whereas 0.5 mL of each strain was used for mixed inoculations. Only sterile water was used for the control plants. Inoculated plants were grown using the same conditions as described above but humidity was increased to 85%. These plants were also covered with a transparent plastic bag for the first three days post inoculation (dpi).

The pathogenicity assay included eight different treatments, namely two sugarcane varieties (LC07-150 and ZZ8) each inoculated with four different inocula: two single strains of *Aaa* (CNGX08 and CNGD05), a mix (1:1) of these two strains, and sterile water (negative control). Thirty plants were inoculated for each treatment and the pathogenicity assay was conducted independently two times. Disease incidence (%) was calculated as follows: (Number of plants with red stripes/Total number of inoculated plants) x 100. Area under disease progress curve (AUDPC) was calculated as follows for each treatment of each of the two inoculation experiments (Shaner, 1977):


$$\text{AUDPC}=\sum_{i=1}^{n}\left[\;\frac{1}{2}\left(X_{i+1}+X_{i}\right)\left(T_{i+1}-T_{i}\right)\;\right]$$

*X_i_* and *X_i_*_+1_ represent the disease incidence at time points *T_i_* and *T_i_*_+1_, respectively; *n* is the total number of time points (0, 3, 6, 9, 12, and 15 dpi). At 15 dpi, three inoculated leaves with red stripe symptoms were used for bacterial isolation and characterization. Bacterial colonies isolated from symptomatic leaves were identified by PCR with *Aaa*-specific primers RS-ITS-F1 and RS-ITS-R1 (Li et al., 2018).

### 2.9 Determination of SA content

Six leaf samples (three from each inoculation experiment) of varieties LC07-150 and ZZ8 inoculated with *Aaa* strains CNGX08 and CNGD05 were collected at 0, 12, 24, 48, and 72 hours post inoculation (hpi). Endogenous SA content of the leaf tissue was determined following the instructions of a plant SA ELISA kit (Beijing Solarbio Science & Technology Co., Ltd. China). Each leaf subsample (0.1 g each representing the six collected samples) was homogenized in 1 mL of 10 mM PBS buffer (pH = 7.4) using a freeze grinder (JXFSTPRP-CL, Shanghai, China). Homogenized leaf tissue was centrifuged at 2500 rpm and 25°C for 20 min and the supernatant was used for determination of SA content by sandwich ELISA and a 450 nm optical density. Three leaf subsamples were analyzed at each time point for each treatment, and three technical replicates were performed per subsample.

### 2.10 Transcript expression analysis of gene *PR-1*

The six leaf samples collected for determination of SA content (section 2.9) were also used to determine relative expression of gene *PR-1.* The transcript expression of gene *PR-1* involved in the SA signaling pathway was determined by RT-qPCR assay using the QuantStudio 3 qPCR System (Applied Biosystems, USA). Total RNA was extracted from the leaf tissue using the TRIzol reagent kit (Invitrogen, USA). The cDNA was synthesized using HiScript II Q RT SuperMix with a qPCR (+gDNA wiper) reverse transcription kit (Vazyme Biotech). The qPCR mix was composed of 10.0 μL of 2× ChamQ Universal SYBR qPCR Master Mix (Vazyme Biotech), 0.4 μL of forward and reverse primers (10 μM), 1.0 μL of cDNA, and sterile high-purity water to obtain a final volume of 20 μL. The qPCR program included denaturation at 95°C for 30 s followed by 40 cycles at 95°C for 10 s, and 60°C for 30 s. The glyceraldehyde 3-phosphate dehydrogenase (GAPDH) gene was used as a reference gene (Zheng et al., 2017). The relative expression of gene *PR-1* was calculated with the 2^-△△CT^ method. Characteristics of the primers is given in **Table S2**. Three leaf subsamples were analyzed at each time point and for each treatment, and three technical replicates were performed per subsample.

### 2.11 Statistical analyses

An analysis of variance (one-way ANOVA) was conducted with AUDPC data obtained for the two sugarcane varieties inoculated with the two strains of *Aaa* (single and mixed inocula). The same type of analysis was performed with each data set of the SA content and of the transcript level of gene *PR-*1 in plants at each time point (0, 12, 24, 48, and 72 h). Duncan’s test was used to identify mean differences at *p* ≤ 0.05 between the treatments. All analyses were conducted with the software SAS version 8.01 (SAS Institute Inc., North Carolina, USA).

## 3 Results

### 3.1 Isolation of bacteria from leaves showing red stripe symptoms

**Figure 1:**
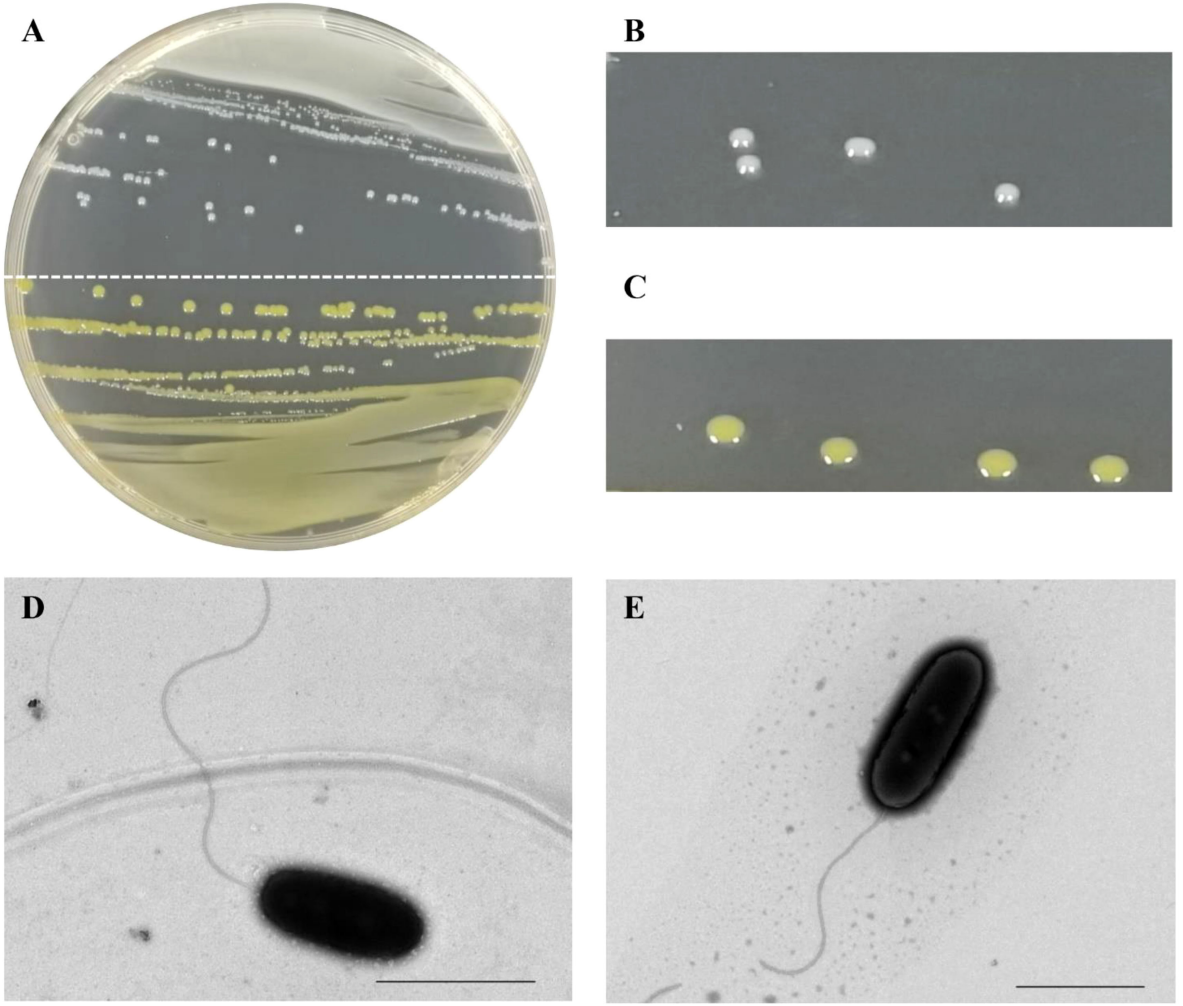
Colony and cell morphology of two strains of *Acidovorax avenae* subsp. *avenae* isolated from sugarcane in China. **(A)** Colonies of strain CNGX08 (white-cream; upper half plate) and strain CNGD05 (yellow; lower half plate) growing on a nutrient agar (NA) medium plate. **(B, C)** Close up view of the colonies of strains CNGX08 and CNGD05, respectively. **(D, E)** Cell morphology of strains CNGX08 and CNGD05 observed by transmission electron microscopy (×10,000), respectively. The scale bar corresponds to 2.0 µm.

Seventeen bacterial strains were isolated on NA medium from the 13 sugarcane leaves (A-M) with typical red stripe symptoms collected from the provinces of Guangxi and Guangdong (**Figure S1**). The color of the colonies varied among these strains (**Figure 1A**). Five leaf samples yielded only white-cream, circular, and translucent colonies whereas another four leaf samples yielded only yellow, circular, and translucent colonies. Colonies of both morphological types were obtained from the remaining four leaf samples. The white-cream colonies grew slower than the yellow colonies as mean size of white-cream colonies was 1.14 mm (± 0.06) vs. 0.91 mm (± 0.06) for yellow colonies after 24 h of growth on NA medium (**Figures 1B, C**). A colony of strain CNGX08 (white-cream) and a colony of CNGD05 (yellow) were taken for observation of bacterial cell morphology by electron microscopy. No bacterial spores were observed in the microscopic preparations and cells of both strains were straight rods with a single polar flagellum (**Figures 1D, E**).

### 3.2 PCR detection and sequence identification of *Aaa* strains

A 454 bp fragment was amplified by PCR from total genomic DNA of the 17 bacterial strains from China using the *Aaa*-specific primers (RS-ITS-F1/RS-ITS-R1) (**Table S1**). Based on the nucleotide sequence of this amplicon, the strains originating from two geographical regions (Guangxi *vs.* Guangdong) or with different morphological types (white-cream *vs.* yellow) were 98.4-100% identical (**Figure S2**). These strains had also 98.2-99.5%, 97.7-98.8%, and 96.2-97.3% sequence identity with *Aaa* strain ATCC 19860 *Aac* strain W6, and *Aaca* strain 30134, respectively. The specific PCR amplification and sequence data suggested that the 17 bacterial strains from sugarcane in China belong to the subspecies *Aaa*.

### 3.3 RFLP analysis of *Aaa* strains based on the 16S-23S rDNA ITS region

The 17 *Aaa* strains from sugarcane in China were divided into three groups based on their *in silico* restriction patterns (I, II, and IIIb) of the 16S-23S rDNA ITS region (454 bp) cut with enzymes *Hin*dIII and *Eco*RI (**Table 1**). Pattern RFLP I consisted of two bands (330 and 124 bp) whereas pattern II had three bands (330, 68, and 56 bp). Two bands, one of 386 bp and the other of 68 bp, formed pattern RFLP IIIb. The RFLP pattern I group included seven *Aaa* strains from the Guangxi province (four with white-cream colonies and three with yellow colonies). RFLP pattern II group contained nine strains from the provinces of Guangxi and Guangdong (four with white-cream colonies and five with yellow colonies). Only strain CNGD01 with white-cream colonies from the province of Guangdong showed RFLP pattern IIIb (**Table 1**).

**Table 1:** *In silico* analysis of RFLP patterns of 17 strains of *Acidovorax avenae* subsp. *avenae* from sugarcane in China based on the internal transcribed spacer (ITS) region of 16S-23S rDNA.

Strain name	Colony color	Size of sequence (bp)	Restriction fragments (bp) obtained after digestion with			Restriction pattern ^a^
			*Hin*dIII	*Eco*RI	*Hin*dIII + *Eco*RI	
CNGX01	white	454	330, 124	454	330, 124	I
CNGX02	yellow	454	330, 124	454	330, 124	I
CNGX03	yellow	454	330, 124	386, 68	330, 68, 56	II
CNGX04	white	454	330, 124	386, 68	330, 68, 56	II
CNGX05	yellow	454	330, 124	454	330, 124	I
CNGX06	white	454	330, 124	454	330, 124	I
CNGX07	yellow	454	330, 124	454	330, 124	I
CNGX08	white	454	330, 124	454	330, 124	I
CNGX09	yellow	454	330, 124	454	330, 124	I
CNGX10	white	454	330, 124	386, 68	330, 68, 56	II
CNGX11	yellow	454	330, 124	386, 68	330, 68, 56	II
CNGD01	white	454	454	386, 68	386, 68	IIIb
CNGD02	white	454	330, 124	386, 68	330, 68, 56	II
CNGD03	white	454	330, 124	386, 68	330, 68, 56	II
CNGD04	yellow	454	330, 124	386, 68	330, 68, 56	II
CNGD05	yellow	454	330, 124	386, 68	330, 68, 56	II
CNGD06	white	454	330, 124	386, 68	330, 68, 56	II

a Classification of restriction patterns as described by Li et al. (2018).

### 3.4 Sequence identity analysis of five housekeeping genes

To further investigate the genetic variability among the 17 strains of *Aaa* from sugarcane in China, pairwise sequence comparisons were carried out with the single sequence of five housekeeping genes (*ugp*B*, pil*T*, lep*A*, trp*B, and *glt*A) and with the concatenated sequence (2,246 nucleotides) of these five genes. When the housekeeping genes were analyzed separately, the lowest sequence identities were found for gene *lep*A (95.0-100%), followed by *ugp*B (95.7-100%) and *glt*A (96.8-100%). Highest sequence identities were observed for genes *trp*B (99.5-100%) and *pil*T (97.9-100%) (**Table S3**). The 17 strains of *Aaa* had 97.8-100% sequence identity among each other and 96.7-97.5% with seven strains of *Aaa* from Argentina when comparisons were based on the concatenated sequence of all five genes. This latter identity was 97.0-97.7%, 94.6-95.4%, and 95.7-96.3% with strain ATCC 19860 of *Aaa*, strain AacW6 of *Aac*, and strain 30134 of *Aaca*, respectively (**Table S3**).

### 3.5 Genotyping of *Aaa* strains by MLST

**Figure 2:**
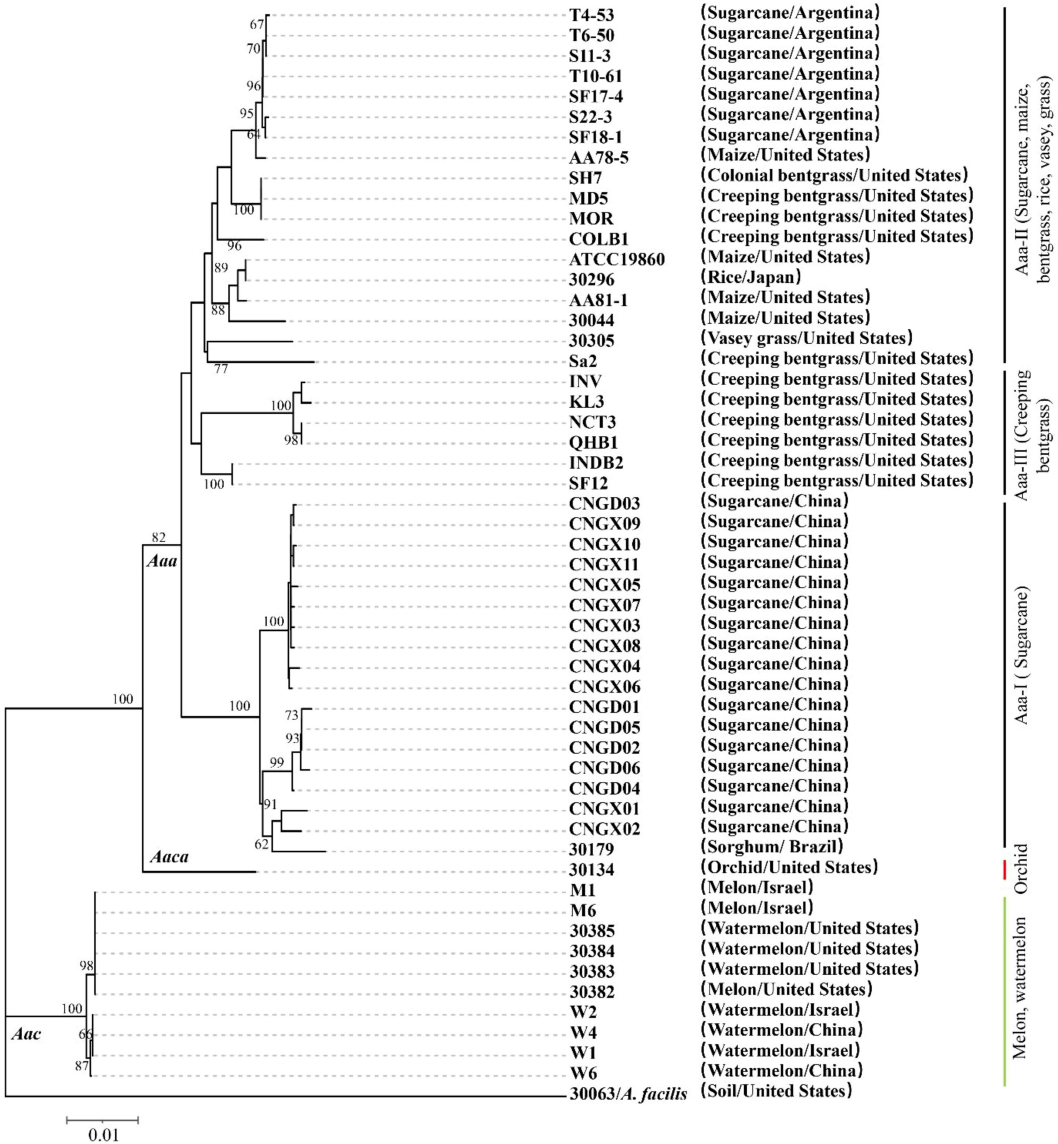
Neighbor-joining phylogenetic tree constructed with the concatenated sequence (2,246 nucleotides) of five housekeeping genes from 42 strains of *Acidovorax avenae* subsp. *avenae* (17 obtained in this study and 25 retrieved from the NCBI library), 10 strains of *A. avenae* subsp. *citrulli* and one strain of *A. avenae* subsp. *cattleyae* retrieved from the NCBI library. Strain 30063 of *A. facilis* was used as an outgroup. The characteristics of all strains are given in **Table S1**. Bootstrap values were determined for 1,000 replications and only bootstrap values >60% are shown at nodes.

To further investigate the evolutionary clustering of *Aaa*, the 17 strains of this subspecies obtained in this study from sugarcane in China were compared to 25 additional strains of *Aaa* (from bentgrass, maize, millet, rice, sorghum, sugarcane, and Vasey grass), 10 strains of *Aac* (from melon and watermelon), and one strain of *Aaca* (from orchid) retrieved from the NCBI library (**Table S1**). MLST conducted with the concatenated sequence of five housekeeping genes (section 3.4) revealed that all *A. avenae* strains were divided at subspecies level into three major evolutionary clades (**Figure 2**). The 10 strains of *Aac* (from China, Israel, and the USA) and strain 30134 of *Aaca* (from the USA) were distributed in two separate evolutionary clades. The 42 strains of *Aaa* forming the third clade were further distributed into three subclades. The 17 strains of *Aaa* from sugarcane obtained in this study and *Aaa* strain 30179 from sorghum in Brazil formed subclade Aaa-I, while seven strains from sugarcane in Argentina and 11 additional strains from bentgrass, maize, rice, and Vasey grass from Japan and the USA grouped in subclade Aaa-II. Six strains of *Aaa* from Creeping bentgrass originating from the USA formed subclade Aaa-III (**Figure 2**). In subclade Aaa-I, the 17 strains of *Aaa* from sugarcane obtained in this study were distributed in three different phylogenetic subgroups.

### 3.6 Pathogenicity of two strains of *Aaa* in sugarcane plants

**Figure 3:**
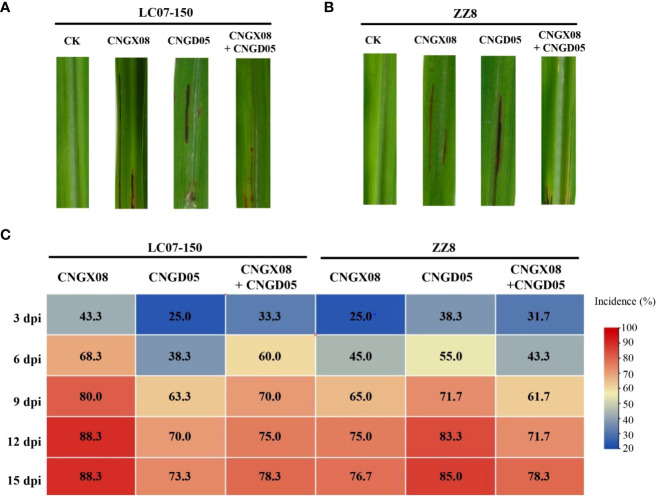
Symptoms and disease incidence of red stripe in two sugarcane varieties (LC07-150 and ZZ8) inoculated with strains CNGX08 and CNGD05 of *Acidovorax* avenae subsp. avenae. **(A)** Red stripe symptoms observed 15 days post inoculation (dpi) in variety LC07-150, CK = water control. **(B)** Red stripe symptoms observed 15 dpi in variety ZZ8, CK = water control. **(C)** Heatmap of disease incidence of inoculated plants from 3-15 dpi.

Red stripe symptoms developed in sugarcane varieties LC07-150 and ZZ8 after single and mixed inoculation with strains CNGX08 and CNGD05 of *Aaa* from China. At 3 dpi, water-green stripes appeared in the middle of the leaf blade and these lesions rapidly turned into red-brown stripes. The sugarcane plants of varieties LC07-150 and ZZ8 inoculated with sterile water remained symptomless (**Figures 3A, B**). The disease incidence of all inoculation treatments with *Aaa* was greater than 20 and 65% at 3 and 12 dpi, respectively (**Figure 3C**). At 15 dpi, lowest incidence (73.3%) was observed for variety LC07-150 inoculated with strain CNGD05 and highest incidence (88.3%) was found for the same variety inoculated with strain CNGX08. After isolation from symptomatic plants, the strains of *Aaa* shared 100% sequence identities of ITS of 16S-23S rDNA and same colony color with inoculated strain.

A significant bacterial strain effect (*P* = 0.0007) but a not significant sugarcane variety effect (*P* = 0.0680) was found based on AUDPC analysis of all inoculation treatments of combined experiments. The variety x strain interaction effect was also not significant (*P* = 0.0591). Strain CNGX08 of *Aaa* was more pathogenic than strain CNGD05 in sugarcane variety LC07-150, while strain CNGD05 was more pathogenic than strain CNGX08 in sugarcane variety ZZ8 (**Table 2**). Strain CNGX08 was also more pathogenic in LC07-150 when inoculated alone than in mixed inoculation with strain CNGD05. The same result was observed for strain CNGD05 in variety ZZ8.

**Table 2:** Analysis of variance of area under disease progress curve (AUDPC*) of two sugarcane varieties inoculated with two strains of *Acidovorax avenae* subsp. *avenae* (*Aaa*).

Treatment	AUDPC**
Sugarcane variety LC07-150 inoculated with *Aaa* strain CNGX08	9.726 a
Sugarcane variety LC07-150 inoculated with *Aaa* strain CNGD05	7.000 c
Sugarcane variety LC07-150 inoculated with *Aaa* strains CNGX08 + CNGD05	8.301 b
Sugarcane variety ZZ8 inoculated with *Aaa* strain CNGX08	7.450 b
Sugarcane variety ZZ8 inoculated with *Aaa* strain CNGD05	8.725 a
Sugarcane variety ZZ8 inoculated with *Aaa* strains CNGX08 + CNGD05	7.425 b

(*AUDPC) values were calculated with disease incidence (percent diseased plants) determined for two separate experiments at 3, 6, 9, 12, and 15 days post inoculation.

(**AUDPC) followed by the same letter are not different at P = 0.05 according to Duncan’s test.

### 3.7 Changes of SA content in sugarcane after infection by *Aaa*

**Figure 4:**
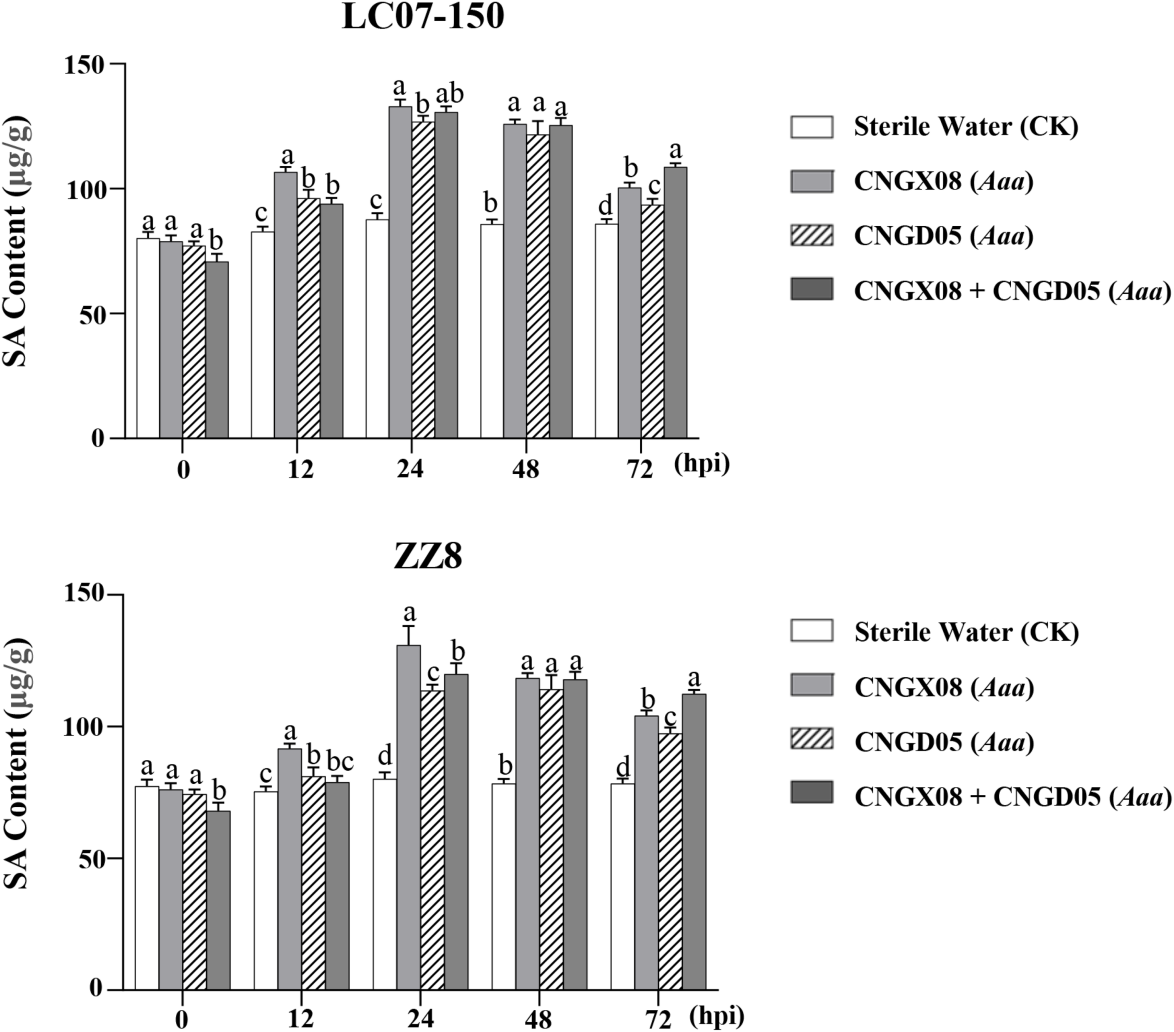
Salicylic acid (SA) content in two sugarcane varieties (LC07-150 and ZZ8) inoculated with strains CNGX08 and CNGD05 of *Acidovorax avenae* subsp. *avenae* (*Aaa*). Inoculated leaf samples were collected 0, 12, 24, 48, and 72 hours post inoculation (hpi). Each vertical bar represents the mean ± standard deviation of three biological and three technical replicates per subsample. At each time point, means with the same letter are not significantly different at *P* = 0.05 according to Duncan’s test.

To investigate whether the SA signal transduction pathway was involved in sugarcane affected by red stripe, the SA content and the expression of the SA-mediated resistance gene (*PR-1*) were determined in sugarcane varieties LC07-150 and ZZ8 after single or mixed inoculation with strains CNGX08 and CNGD05 of *Aaa.* Similar changes in SA content were observed in the two sugarcane varieties 0-72 hpi, regardless of the bacterial inoculum (**Figure 4**). When compared to the control plants inoculated with water, the SA content increased in varieties LC07-150 and ZZ8 after all *Aaa* treatments. Highest SA increases (40-65%) were observed in these varieties at 24-48 hpi. Significant differences between *Aaa* treatments were observed at some time points but they were not always consistent throughout the 12-72 hpi. Nevertheless, SA content was greater in varieties LC07-150 and ZZ8 inoculated with strain CNGX08 of *Aaa* than with strain CNGD05 at 12, 24, and 72 hpi (**Figure 4**).

### 3.8 Expression of gene *PR-1* in sugarcane infected by *Aaa*

**Figure 5:**
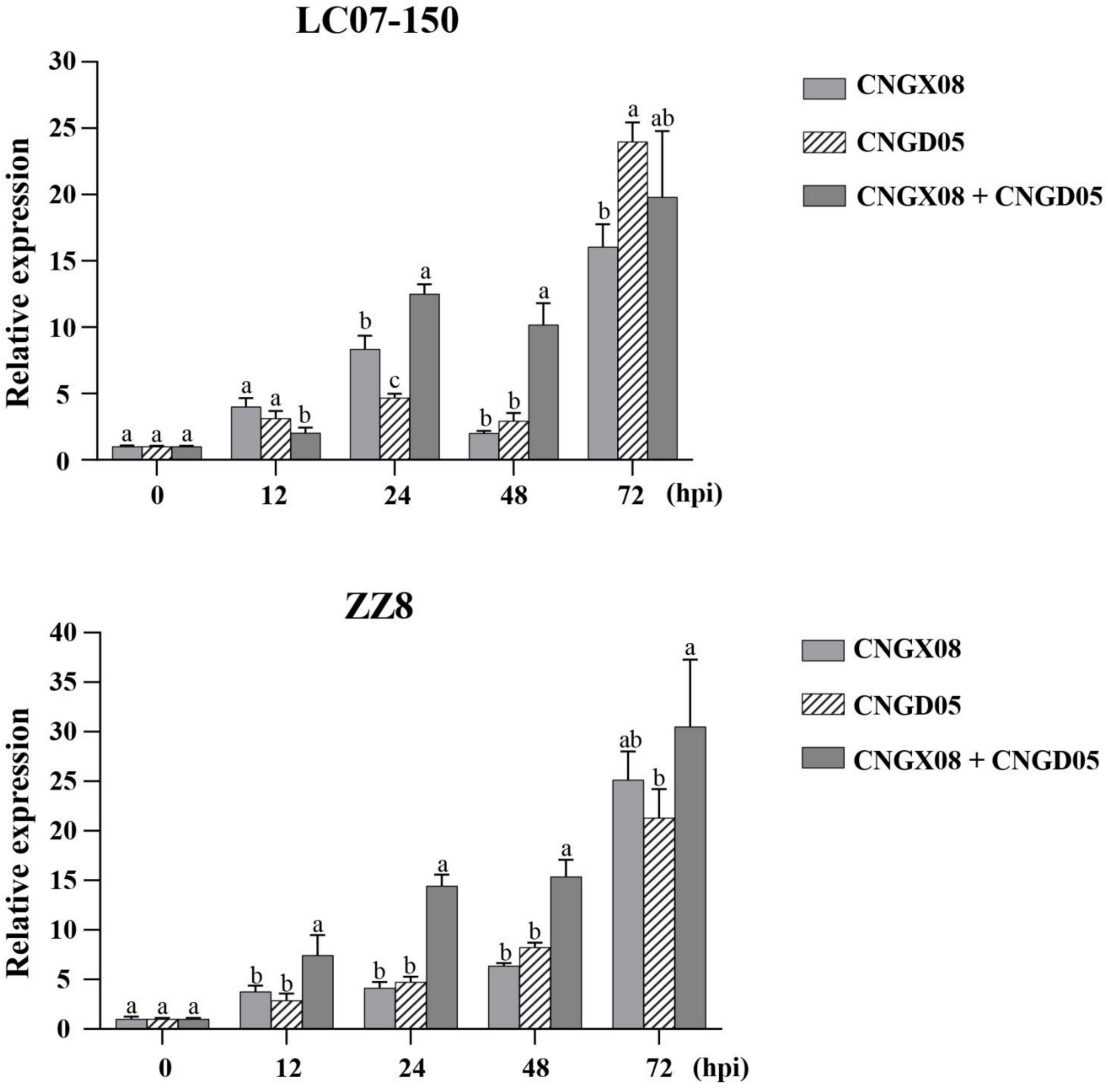
Relative expression of pathogenesis-related protein 1 (PR-1) gene in two sugarcane varieties (LC07-150 and ZZ8) inoculated with strains CNGX08 and CNGD05 of *Acidovorax avenae* subsp. *avenae* (*Aaa*). Inoculated leaf samples were collected 0, 12, 24, 48, and 72 hours post inoculation (hpi). Each vertical bar represents the mean ± standard deviation of three biological and three technical replicates per subsample. At each time point, means with the same letter are not significantly different at *P* = 0.05 according to Duncan’s test.

Expression of gene *PR-1* involved in the SA signal transduction pathway was significantly upregulated in varieties LC07-150 and ZZ8 inoculated with the two strains of *Aaa* (CNGX08 and CNGD05), as compared to control plants inoculated with water only (**Figure 5**). Significant increases of *PR-1* expression were observed for single and mixed inocula as soon as 12 hpi, but greatest expression was found at 72 hpi. At his time point, *PR-1* transcript levels had increased 16-23 times in variety LC07-150 after single or mixed inoculation with strains CNGX08 and CNGD05 of *Aaa.* A 21-30-time increase was observed for the same strains in variety ZZ8. In each infected sugarcane variety, relative expression of gene *PR-1* differed among strains at one time point or another but no consistent pattern was observed, except at 24 and 48 hpi. At these two time points, relative expression of gene *PR-1* was higher in plants of the two varieties inoculated simultaneously with strains CNGX08 and CNGD05 than in plants inoculated with the single strains of the pathogen.

## 4 Discussion

Variation in color of *Aaa* colonies has been reported, especially when this bacterial subspecies was grown on different agar media. For example, colonies of *Aaa* from sugarcane were off-white on potato dextrose agar (PDA) (Zia-ul-Hussnain et al., 2011), yellow or beige on yeast extract-dextrose-calcium carbonate (YDC) (Zia-ul-Hussnain et al., 2011; Fontana et al., 2013; Bertani et al., 2021), yellow-cream on yeast peptone glucose agar (YPGA) (Girard et al., 2014), white-cream on NA medium (Fontana et al., 2013; Li et al., 2018; Bertani et al., 2021), and grayish-white on Wilbrinks agar medium (Hernández-Juárez et al., 2021). However, only 35-50% of white-cream colonies isolated from sugarcane in Argentina on NA medium were positive by PCR with *Aaa*-specific primers Oaf1/Oar1 (Fontana et al., 2013; Bertani et al., 2021). PCR-negative colonies and other yellow-colored colonies grown on NA or YDC media belonged to other bacterial species of the *Erwinia* and *Pantoea* genera.

Colonies of *A. avenae* are usually white-cream and do not produce pigments on NA medium at optimal growth temperature of 30-35°C (Willems and Gillis, 2015). In our study based on microbiological, molecular, and pathogenicity assays, two types of colored colonies (yellow and white) growing on NA medium were identified as the causal agents of sugarcane red stripe in China. A bias of color caused by the medium composition was excluded because both colored colonies were observed on the same plates and under the same environmental conditions. Furthermore, only one color type of colony was reisolated from plants inoculated with pure cultures of two strains, namely white-cream colored strain CNGX08 and yellow-colored CNGD05. No difference in cell morphology (shape of bacteria) was found between these two strains that differed from each other based on growth rate on NA medium (CNGX08 grew slower than CNGD05), RFLP profile (I for CNGX08 and II for CNGD05), and phylogenetic subgrouping in the Aaa-I clade. The genetic mechanism for color variation in *Aaa* remains to be investigated. Occurrence of two colored types of *Aaa* in some infected plants also revealed that red stripe can be caused by a mix of strains. Mixed plant infections with diverse strains of a microorganism (including pathogens) are common in crops, especially in sugarcane that is propagated vegetatively by stalk cuttings (He et al., 2022).

Genetic diversity of *Aaa* strains in sugarcane-growing countries was previously unraveled by various molecular typing methods such as RAPD (Fontana et al., 2013), PCR-based RFLP (Li et al., 2018), MLST (Fontana et al., 2019), rep-PCR and AFLP (Bertani et al., 2021). Among these methods, MLST is very powerful to analyze the taxonomic structure, evolutionary processes, and population speciation of bacteria, including *Aaa* (Rong and Huang, 2014; Fontana et al., 2019). In our study, the MLST analysis revealed that all *Aaa* strains hosted by various plant species worldwide formed a distinct and separate clade from strains of *Aac* (hosted by watermelon and melon) and *Aaca* (hosted by orchid) at the subspecies level. Additionally, the strains of *Aaa* isolated from sugarcane in Argentina and China were distributed in two different evolutionary subclades. The subclade of sugarcane strains from Argentina also contained strains from other *Poaceae* such as bentgrass, maize, rice, sorghum, and Vasey grass, whereas the subclade of sugarcane strains from China only included one strain from sorghum in Brazil. Similarly, several strains of *Aaa* from bentgrass in the USA were closer to strains of *Aaa* from maize or rice rather than to other strains collected from bentgrass in the same country (Zeng et al., 2017). These data suggested that *A. avenae* strains exhibit clonal behavior, geographical and host specificity to a certain extent, as previously reported (Yan et al., 2017; Fontana et al., 2019; Bertani et al., 2021). Among seven housekeeping genes used for a MLST study in Argentina, gene *lep*A exhibited greatest variability for strains of *Aaa* from sugarcane (Fontana et al., 2019).

Besides the 16S rDNA, the ITS region of 16S–23S rDNA is also very useful for bacterial and fungal species or strain classification because PCR amplification and sequencing of this region provides specific and accurate data using universal primers (Ji et al., 2017; Li et al., 2018). Consequently, this method has also been widely applied to identify and differentiate *A. avenae* at species, subspecies, and strain levels (Schaad et al., 2008; Song et al., 2003; Li et al., 2018). For example, Song et al. (2003) developed the species-specific primers Oafl/Oarl for rapid identification of *A. avenae* strains from all hosts, whereas the *citrulli*-specific primers Aacf2/Aacr2 are for specific identification of subsp. *citrulli*. Schaad et al. (2008) reported that sequence identities of the ITS of the 16S–23S rDNA were high (97.3-99.0%) among strains of *A. avenae* from various hosts, whereas the other three plant pathogenic species (*A. konjaci*, *A. anthurii*, and *A. valerianellae*) shared lower identities (81.8–87.8%) with *A. avenae* strains. Li et al. (2018) demonstrated that these nucleotide sequences had also different sizes (from 436 to 454 bp) and that their identities diverged from 89.2 to 100% among *Aaa* strains infecting sugarcane from China and *A. avenae* strains infecting other plants worldwide. Furthermore, seven RFLP profiles based on the ITS region of 16S-23S rDNA were reported for strains of *Aaa* from sugarcane in China, among which profiles I, II, IIIb, and IVa were reported in the provinces of Guangxi and Guangdong (Li et al., 2018). The most recent strains collected in our study from the same provinces belonged to RFLP profiles I, II, and IIIb, thus confirming previous results.

No relationship was found between the three RFLP profiles of the 17 strains of *Aaa* obtained herein and the geographical origin, host variety, and colony color. Similarly, no correlation was found between the geographical origin and the sampling year of strains of *Aaa* from Argentina using MLST (Fontana et al., 2019). Genetic diversity of *Aaa* identified by rep-PCR was not related either with sugarcane genotype, ratoon cycle, tissue type, field fertilization, or year of sampling (Bertani et al., 2021). It has been hypothesized that dispersal rates of *Aaa* are too high to maintain spatially structured populations within a limited geographic distance and in a single host (Zeng et al., 2017; Bertani et al., 2021). Nevertheless, specific genetic populations of *Aaa* were identified by MLST in China in this study and previously in Argentina. To confirm this, further studies need to be conducted with a more extensive collection of strains of the pathogen from additional locations affected by sugarcane red stripe.

After inoculation of two sugarcane varieties susceptible to red stripe, strains CNGX08 (white-cream colonies) and CNGD05 (yellow colonies) of *Aaa* induced development of disease symptoms and disease incidence was high (≥70%) 15 days after inoculation. Similarly, high disease incidence (53-70%) was reported seven days post inoculation of variety FN41 with *Aaa* strain SC-026 from China (Li et al., 2018). Creeping bentgrass inoculated with strain MSU4 of *Aaa* exhibited up to 68% of leaf necrosis two weeks after inoculation (Giordano et al., 2012). Variation in pathogenicity among strains of *Aaa* is currently poorly known, although different levels of aggressiveness in a resistant sugarcane variety were found among isolates of this pathogen in Argentina (Bertani et al., 2021).

Strain CNGX08 of *Aaa* from China was more pathogenic than strain CNGD05 in one sugarcane variety (LC07-150) whereas strain CNGD05 was more pathogenic than CNGX08 in the other variety (ZZ8). This is strong evidence for variation in pathogenicity and possible occurrence of races within *Aaa* as the variety x strain interaction effect was almost significant (*P* = 0.0591). The genetic basis for resistance of sugarcane to red stripe is currently unknown. Besides specific features of the pathogen involved in pathogenicity variation, the host cytoplasm may also play an important role in the sugarcane response to a specific strain of *Aaa* as strains CNGX08 and CNGD05 were more pathogenic in the sugarcane variety from which they were originally isolated. Use of additional inoculation experiments and different sugarcane varieties might be necessary to prove these hypotheses. In the host variety in which their incidence was highest, strains CNGX08 and CNGD05 of *Aaa* were also more pathogenic in single inoculations than in mixed inoculations, suggesting a negative interaction between the two strains. No significant difference in pathogenicity was found for *Cylindrocarpon destructans* when single and mixed inocula of this root pathogen of oak (*Quercus* spp.) were compared (Sánchez et al., 2002). In contrast, the nonpathogenic *hrcC* mutant of *Xanthomonas campestris* pv. *vesicatoria* 85-10::hrpA22 multiplied in pepper leaves when it was mixed with the pathogenic wild type strain, thus indicating a positive effect of this latter strain in mixed inoculation (Keshavarzi et al., 2004). When tested alone, lineage IV of *Cephalosporium maydis* from Egypt was the most virulent of four clonal lineages toward greenhouse-grown maize. However, this lineage was the least competitive in susceptible maize clones inoculated with mixtures of all four lineages of the pathogen (Zeller et al., 2002). Further investigations are needed to understand the interactions among strains of *Aaa*, especially in diseased plants infected by the two differently colored types of the pathogen.

SA and the SA-mediated signal transduction pathway are major players in plant immunity by mediating local and systemic immune responses against pathogen invasion (Seyfferth and Tsuda, 2014; Khan et al., 2022). SA levels are usually elevated in plants upon pathogen infections, thus promoting massive transcriptional reprogramming including increased expression of gene *PR-1* (Chu et al., 2020; Chu et al., 2022; Khan et al., 2022). SA and Jasmonic (JA) treatments induced tolerance to *Aaa* infection in creeping bentgrass (Liu et al., 2018). In our study, the two strains of *Aaa* induced significant increases of endogenous SA content and expression of gene *PR-1* in infected sugarcane, especially at 24-72 hpi. Increased content of SA and transcript expression of *PR-1* was observed in sugarcane after inoculation with two strains (XaCN51 and XaCN24) of *X. albilineans* differing in pathogenicity (Zhao et al., 2022). Moreover, plants infected with the high pathogenic strain of *X. albilineans* (XaCN51) maintained a lower content of SA and a lower expression level of SA-medicated gene *PR-1* as compared to plants infected with the low pathogenic strain of the pathogen (XaCN24). In contrast to the study on *X. albilineans*, the content of SA and the expression level of SA-mediated gene *PR-1* in *Aaa* infected sugarcane were not associated with disease level caused by the two strains of the pathogen (CNGX08 and CNGD05). One reason could be the high expression of disease symptoms for the two strains even if their disease incidences were significantly different. Another reason could be that the SA signaling pathway plays a minor role in the defense response against *Aaa* as compared to other signaling pathways. These hypotheses remain to be explored. Significant difference in disease incidence and in the SA plant response between the two strains of *Aaa* from China (single and mixed infections) suggested that colony color could be associated with pathogenicity of the red stripe pathogen of sugarcane. This hypothesis needs to be investigated by testing a larger number of strains of each color type and by functional genomics of genes involved in colony color.

## 5 Conclusion

In this study, we demonstrated that at least two strains of *Aaa* with different colony colors (i.e. white-cream and yellow) cause red stripe of sugarcane in China. Based on MLST and PCR-based RFLP data, plant species and geographical origin at country level appeared to contribute to genetic diversity of *Aaa* populations rather than host varieties in a given small geographical environment. SA and gene *PR-1* that are usually involved in resistance to biotic stresses are also produced/expressed in a susceptible interaction leading to disease. Additional studies are needed to elucidate the significance of color variation in *Aaa* and the impact of the genetic diversity of this pathogen in red stripe epidemics of sugarcane and other crops.

## Data availability statement

The original contributions presented in the study are included in the article/**Supplementary Material**. Further inquiries can be directed to the corresponding authors.

## Author contributions

Conceptualization, PR and S-JG. Writing—original draft preparation, J-YZ and JC. Writing—review and editing, PR and S-JG. Data curation, Z-TH and JL. Experiments preformation, J-YZ, JC, and H-YF. Supervision, funding acquisition, and project administration, S-JG. All authors contributed to the article and approved the submitted version.
