# Supplementary material for: Genetic and morphological variants of Acidovorax avenae subsp. avenae cause red stripe of sugarcane in China
Source: Front Plant Sci. 2023 Feb 6;14:1127928. doi: 10.3389/fpls.2023.1127928 (PMC9939834; doi:10.3389/fpls.2023.1127928)
Supplement: Supplementary file 1 [file DataSheet_1.docx]

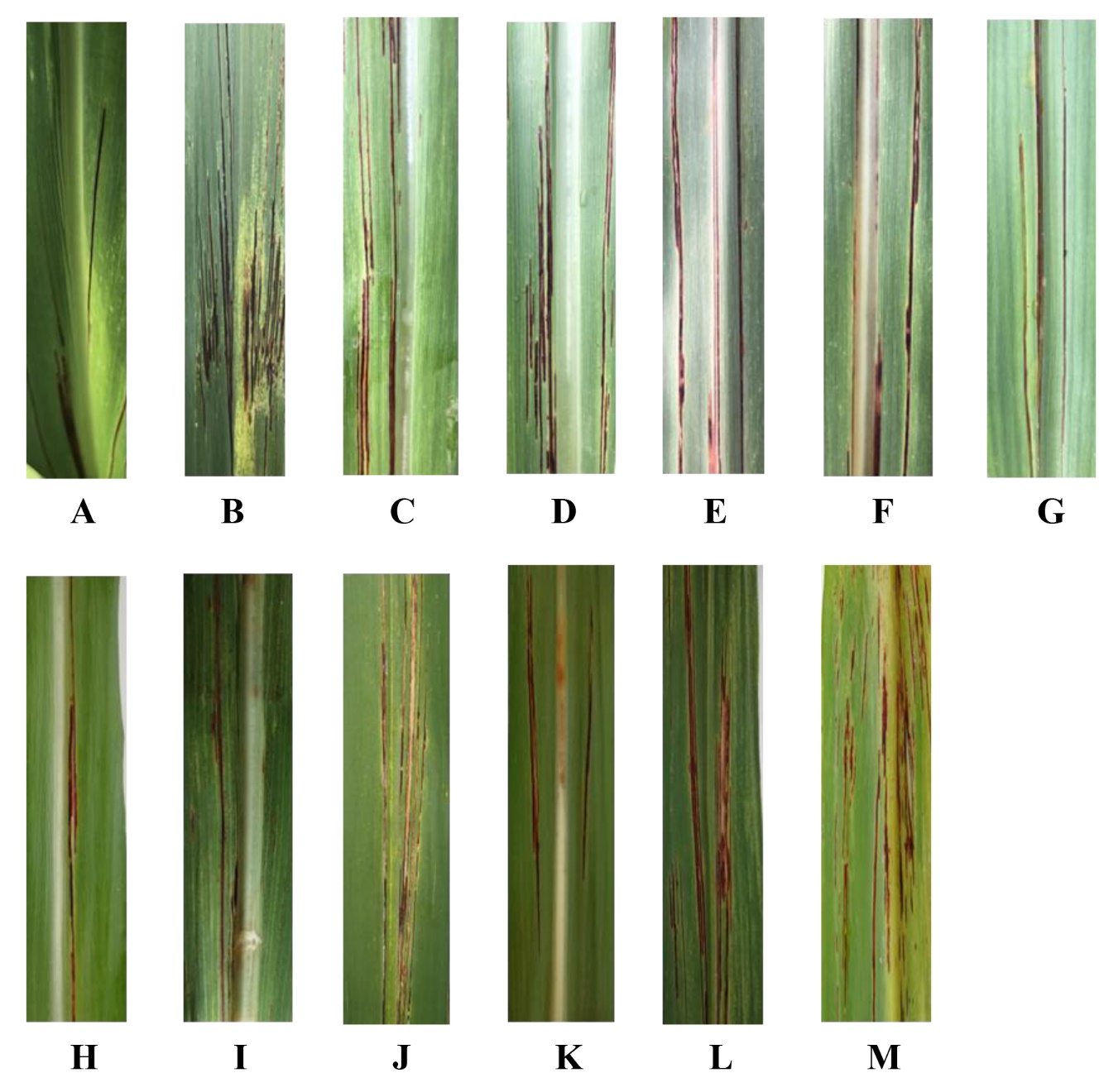


**Figure S1** Symptoms of red stripe on 13 field leaf samples (A-M) of sugarcane collected in China. A: Unknown sugarcane variety (source of strains CNGX01 and CNGX02 of *Acidovorax avenae* subsp. *avenae*); B: Unknown variety (strain CNGX03); C: Variety FN 38 (strains CNGX04 and CNGX05); D: Variety GT 58 (strains CNGX06 and CNGX07); E: Variety LC07-150 (strain CNGX08); F: Variety LC07-150 (strain CNGX09); G: Variety LC07-150 (strains CNGX10 and CNGX11); H: Variety YT00236 (strain CNGD01); I: Variety ZT8 (strain CNGD02); J: Variety ZZ8 (strain CNGD03); K: Variety YZ15-505 (strain CNGD04); L: Variety ZZ 8 (strain CNGD05); M: Variety YT83-27 (strain CNGD04).


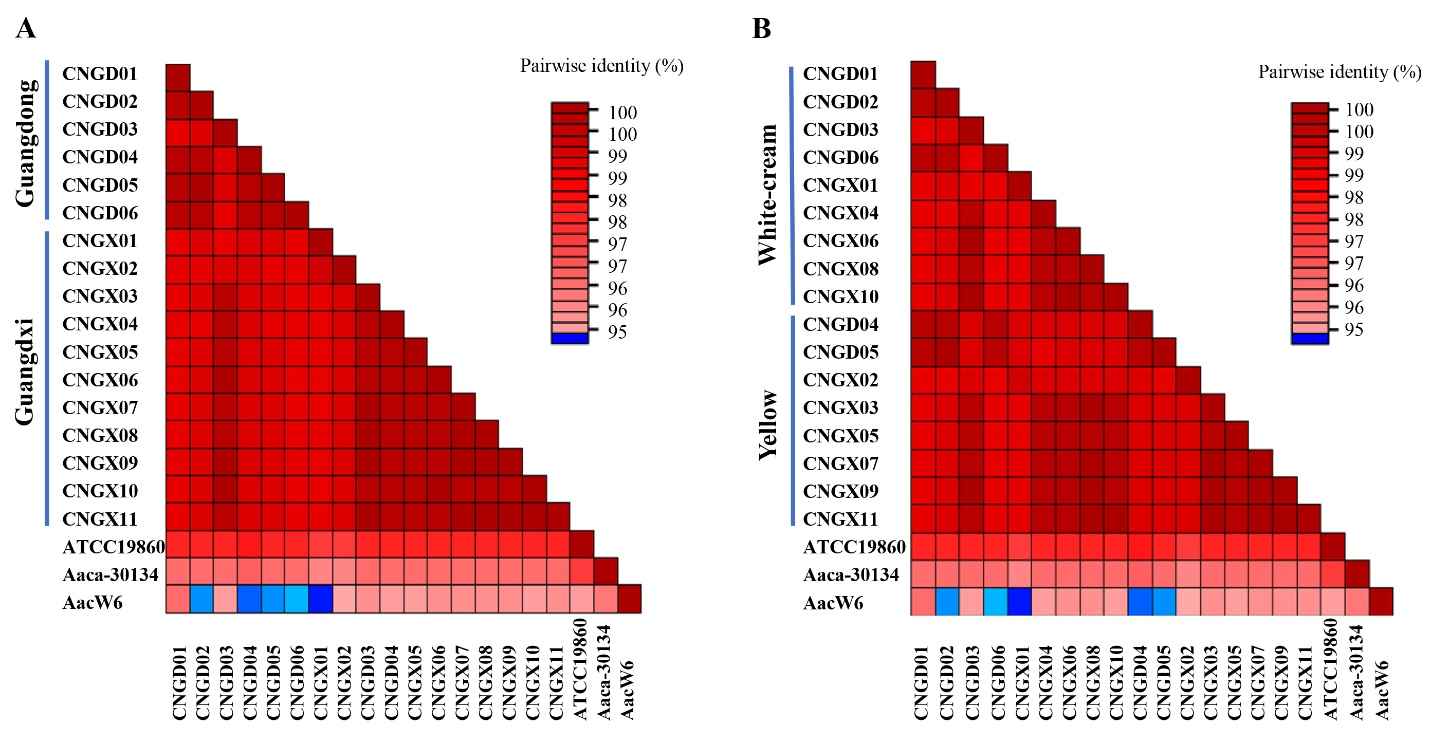


**Figure S2** Heatmap of nucleotide sequence identities of 17 strains of *Acidovorax avenae* subsp. *avenae* (*Aaa*) from sugarcane in China and three reference strains of *Acidovorax avenae* subspecies (strain ATCC19860 of *Aaa*; strain AacW6 of *A. avenae* subsp. *citrulli*, and strain 30134 of *A. avenae* subsp. *cattleyae*). The heatmap was based on the internally transcribed spacer (ITS) sequences of the 16S-23S rDNA region. A: Heatmap differentiating the 17 strains of *Aaa* from China by geographical origin (Guangxi and Guangdong provinces); B: Heatmap differentiating the 17 strains of *Aaa* from China by colony color (white-cream and yellow).

**Table S1** Characteristics of the strains of *Acidovorax* used in this study.

| No. | Species*/Strain | Host variety | Sampling location | Sampling date | Colony color |
| --- | --- | --- | --- | --- | --- |
| 1 | *Aaa*/CNGX01 | Sugarcane | China: Nanning, Guangxi | 2021/05 | white |
| 2 | *Aaa*/CNGX02 |  |  |  | yellow |
| 3 | *Aaa/*CNGX03 | Sugarcane | China: Nanning, Guangxi | 2021/05 | yellow |
| 4 | *Aaa*/CNGX04 | Sugarcane variety FN38 | China: Nanning, Guangxi | 2021/05 | white |
| 5 | *Aaa*/CNGX05 |  |  |  | yellow |
| 6 | *Aaa*/CNGX06 | Sugarcane variety GT58 | China: Nanning, Guangxi | 2021/05 | white |
| 7 | *Aaa*/CNGX07 |  |  |  | yellow |
| 8 | *Aaa*/CNGX08 | Sugarcane variety LC07-150 | China: Nanning, Guangxi | 2021/05 | white |
| 9 | *Aaa*/CNGX09 | Sugarcane variety GT46 | China: Nanning, Guangxi | 2021/05 | yellow |
| 10 | *Aaa*/CNGX10 | Sugarcane variety LC07-150 | China: Nanning, Guangxi | 2021/05 | white |
| 11 | *Aaa*/CNGX11 |  |  |  | yellow |
| 12 | *Aaa*/CNGD01 | Sugarcane variety YT00-236 | China: Zhanjiang, Guangdong | 2021/06 | white |
| 13 | *Aaa*/CNGD02 | Sugarcane variety ZT8 | China: Zhanjiang, Guangdong | 2021/06 | white |
| 14 | *Aaa*/CNGD03 | Sugarcane variety ZZ8 | China: Zhanjiang, Guangdong | 2021/07 | white |
| 15 | *Aaa*/CNGD04 | Sugarcane variety YZ15-505 | China: Zhanjiang, Guangdong | 2021/07 | yellow |
| 16 | *Aaa*/CNGD05 | Sugarcane variety ZZ8 | China: Zhanjiang, Guangdong | 2021/07 | yellow |
| 17 | *Aaa*/CNGD06 | Sugarcane variety YT83-27 | China: Shaoguan, Guangdong | 2021/07 | white |
| 18 | *Aaa*/SF17-4 | Sugarcane | Argentina |  | white |
| 19 | *Aaa*/S11-3 | Sugarcane | Argentina |  | white |
| 20 | *Aaa*/T10-6 | Sugarcane | Argentina |  | white |
| 21 | *Aaa*/S22-3 | Sugarcane | Argentina |  | white |
| 22 | *Aaa*/SF18-1 | Sugarcane | Argentina |  | white |
| 23 | *Aaa*/T4-53 | Sugarcane | Argentina |  | white |
| 24 | *Aaa*/T6-50 | Sugarcane | Argentina |  | white |
| 25 | *Aaa*/ATCC19860 | Maize | USA |  |  |
| 26 | *Aaa*/30296 | Rice | Japan |  |  |
| 27 | *Aaa*/30179 | Sorghum | Brazil | 1987 |  |
| 28 | *Aaa*/30044 | Millet | Nigeria | 1984 |  |
| 29 | *Aaa*/KL3 | Creeping bentgrass | USA/Ohio | 2010 |  |
| 30 | *Aaa*/INV | Creeping bentgrass | USA/Ohio | 2010 |  |
| 31 | *Aaa*/QHB1 | Creeping bentgrass ‘Penn G2’ | USA/North Carolina | 2009 |  |
| 32 | *Aaa*/NCT3 | Creeping bentgrass ‘Penn G2’ | USA/North Carolina | 2011 |  |
| 33 | *Aaa*/Sa2 | Creeping bentgrass ‘Alpha’ | USA/Pennsylvania | 2011 |  |
| 34 | *Aaa*/AA78-5 | Maize | USA/Georgia | 1978 |  |
| 35 | *Aaa*/AA81-1 | Maize | USA/Georgia | 1981 |  |
| 36 | *Aaa*/COLB1 | Creeping bentgrass ‘Penn A4’ | USA/Texas | 2010 |  |
| 37 | *Aaa*/SF12 | Creeping bentgrass | USA/New York | 2011 |  |
| 38 | *Aaa*/INDB2 | Creeping bentgrass | USA/Indiana | 2010 |  |
| 39 | *Aaa*/MOR | Creeping bentgrass | USA/Ohio | 2010 |  |
| 40 | *Aaa*/SH7 | Colonial bentgrass ‘Greenwich’ | USA/Rhode Island | 2010 |  |
| 41 | *Aaa*/MD5 | Creeping bentgrass | USA/Illinois | 2010 |  |
| 42 | *Aaa*/30305 | Vasey grass | USA | 1997 |  |
| 43 | *Aac*/W1 | Watermelon | Israel | 2002 |  |
| 44 | *Aac*/W2 | Watermelon | Israel | 2003 |  |
| 45 | *Aac*/W4 | Watermelon | China | 2009 |  |
| 46 | *Aac*/W6 | Watermelon | China | 2009 |  |
| 47 | *Aac*/M1 | Melon | Israel | 2000 |  |
| 48 | *Aac*/M6 | Melon | Israel | 2002 |  |
| 49 | *Aac*/30382 | Melon | USA | 1978 |  |
| 50 | *Aac*/30383 | Watermelon | USA | 1978 |  |
| 51 | *Aac*/30384 | Watermelon | USA | 1978 |  |
| 52 | *Aac*/30385 | Watermelon | USA | 1978 |  |
| 53 | *Aaca*/30134 | Orchid | USA |  |  |
| 54 | *Af*/30063 | Soil | USA |  |  |

**Aaa* = *Acidovorax avenae* subsp. *avenae*; *Aac* = *A. avenae* subsp. *citrulli*; *Xaca = A. avenae* subsp. *cattleyae; Af = A. facilis.*

**Table S2** Primers used in this study for PCR and RT-qPCR assays, and amplification and cloning of five housekeeping genes of *Acidovorax avenae* subsp. *avenae*.

| Target gene/region | Primer name | Sequence (5’→3’) | Fragment size (bp) | Reference |
| --- | --- | --- | --- | --- |
| *ugp*B | ugpB-F | TGAAGGAAATCTCGGTCGTC | 444 | Feng et al. 2009 |
|  | ugpB-R | CTTGACGTCGTTGCTGAAGA |  |  |
| *pil*T | pilT-F | GAGTACATCTGCGCCACCTT | 398 | Feng et al. 2009 |
|  | pilT-R | GAATACGGGCACATCCTGAC |  |  |
| *lep*A | lepA-F | GATCGACACGCCCGGACAC | 489 | Feng et al. 2009 |
|  | lepA-R | TGATGTAGCCCACCTCGCC |  |  |
| *trp*B | trpB-F | GCCACTTCGGCCGCTATG | 434 | Feng et al. 2009 |
|  | trpB-R | CCTCGTTGAGCGCATCCTT |  |  |
| *glt*A | gltA-F | GAAGTCCACGTTCGGGTAGA | 481 | Feng et al. 2009 |
|  | gltA-F | TACATGTACCCGCAGAACCA |  |  |
| ITS ^a^ | RS-ITS-F1 | AGACCCACCAAATCTTCCG | 454 | Li et al. 2018 |
|  | RS-ITS- R1 | GACATCTCCGCTTTCTTTCAAG |  |  |
| *ScPR1* | q-ScPR1-F | CCTATGGCGGTGGAAGATGC | 110 | Chu et al. 2022 |
|  | q-ScPR1-R | TCTGTGGTGTCTGCTTGTGC |  |  |
| *ScGAPDH* | GAPDH-F | CACGGCCACTGGAAGCA | 110 | Zheng et al. 2017 |
|  | GAPDH-R | TCCTCAGGGTTCCTGATGCC |  |  |

^a^ 16S–23S rDNA internally transcribed spacer region.

**Table S3** Comparison of the nucleotide sequence identities of 17 strains of *Acidovorax avenae* subsp. *avenae* (*Aaa*) from sugarcane in China, seven strains of *Aaa* from Argentina, and three reference strains of *Acidovorax avenae* subspecies [strain ATCC19860 of *Aaa*; strain AacW6 of *A. avenae* subsp. *citrulli* (*Aac*), and strain 30134 of *A. avenae* subsp. *cattleyae* (Aaca)]. Comparisons were conducted with the sequences of five housekeeping genes.

| Housekeeping gene | Sequence identity (%) ^a^ | | | | |
| --- | --- | --- | --- | --- | --- |
|  | Among 17 strains of *Aaa* from China | Comparison of 17 strains of *Aaa* from China with | | | |
|  |  | 7 strains of *Aaa* from Argentina | Reference strain ATCC19860 (*Aaa*) | Reference strain AacW6 (*Aac*) | Reference strain 30134 (*Aaca*) |
| *ugp*B | 95.7-100 | 95.2-97.7 | 95.7-97.7 | 87.8-90.7 | 95.4-97.5 |
| *pil*T | 97.9-100 | 97.7-98.7 | 96.4-96.9 | 96.7-97.4 | 95.7-96.4 |
| *lep*A | 95.0-100 | 96.1-96.9 | 96.7-97.1 | 95.2-95.7 | 95.2-95.7 |
| *trp*B | 99.5-100 | 97.2-97.6 | 97.2-97.6 | 95.1-95.6 | 94.9-95.3 |
| *glt*A  All 5 genes | 96.8-100  97.8-100 | 96.6-97.5  96.7-97.5 | 97.0-97.9  97.0-97.7 | 97.2-97.9  94.6-95.4 | 96.8-97.5  95.7-96.3 |

^a^ Strains of *Aaa* from Argentina = T4-53, T6-50, S11-3, T10-61, SF17-4, S22-3, and SF18-1 (Fontana *et al*. 2013 and 2019).
